# Caries inhibition with CO_2_-laser during orthodontic treatment: a study protocol for a randomized split-mouth controlled clinical trial

**DOI:** 10.1186/s13063-022-06117-y

**Published:** 2022-03-12

**Authors:** Ani Bozhidarova Belcheva, Maria Petrova Shindova

**Affiliations:** Department of Paediatric Dentistry, Faculty of Dental Medicine, Medical University of Plovdiv, Plovdiv, Bulgaria

**Keywords:** Carbon dioxide laser, Fluoride, Orthodontic treatment, Caries inhibition

## Abstract

**Introduction:**

White spot lesions associated with orthodontic treatment are a common problem. Recent studies reported increased resistance to acid demineralization of enamel after sub-ablative CO_2_-laser irradiation in a combination with fluoride application. The aim of the study is to assess the efficacy of CO_2_-laser in combination with a fluoride varnish in the prevention, severity, and extent of white spot lesions during orthodontic treatment with fixed appliances.

**Methods and analysis:**

This is a protocol for a randomized, split-mouth controlled, clinical trial. The participants will be children aged 12–18 years at high caries risk, requiring fixed orthodontic treatment. The vestibular surfaces of maxillary anterior teeth of eligible patients will be exposed to CO_2_-laser irradiation in combination with fluoride therapy and fluoride therapy alone followed by bonding of orthodontic brackets. The patients will be recalled 6 and 12 months post-irradiation. Outcome measures will be visual examination with International Caries Detection and Assessment System criteria and SoproLife fluorescence. Data will be analyzed by Student’s *t* test for paired samples and proportional odds logistic regression model, *p*<0.05.

**Ethics and dissemination:**

The study protocol has been approved by the Committee for Scientific Research Ethics, Medical University-Plovdiv, Bulgaria (Reference number P-605/27.03.2020, Protocol of approval No. 2/01.04.2021) and registered on a publicly accessible database. This research received institutional funding from the Medical University–Plovdiv, Bulgaria. The results will be presented through peer-reviewed publications and conference presentations.

**Trial registration:**

ClinicalTrials.gov NCT04903275. Registered on June 2021.

**Supplementary Information:**

The online version contains supplementary material available at 10.1186/s13063-022-06117-y.

## Introduction

### Background and rationale

Orthodontic treatment leads to preferential growth of cariogenic bacteria due to the increase of biofilm accumulation, difficult oral hygiene, reduction of the self-cleaning mechanisms of the oral cavity [1, 2]. Orthodontic appliances, such as fixed braces, automatically place the patient at least at a high or extremely high risk of oral diseases [3]. In a recent study from 2020, Pinto et al. found that individuals undergoing fixed orthodontic therapy for 1 year had a significantly higher incidence and increase of active caries lesions than those without a fixed appliance [2]. The early development of the initial caries lesion in the enamel starts underneath a relatively intact superficial layer of the enamel surface. As a result of the process of demineralization, tissue defects appear in the enamel that correlates to the porous white opacities seen clinically. “White spot lesion” is the most commonly used term in dental literature to describe the subsurface enamel porosity as a result of carious demineralization [4].

The carbon dioxide laser (CO_2_-laser) is reported to increase the acid resistance of enamel due to changes in the hydroxyapatite crystals and the melting and recrystallization of the carbonated hydroxyapatite crystals [5, 6]. Several studies have suggested that CO_2_-laser is the most effective in the prevention of caries [6–10]. Furthermore, the results of recent studies reported increased resistance to acid demineralization of the enamel after sub-ablative CO_2_-laser irradiation in a combination of fluoride application [5, 11–16]. The synergistic mechanism of laser with fluoride is due to the removal of the organic matrix that would render a greater surface area for an increased fluoride uptake both superficially by forming calcium fluoride (CaF_2_) and in its crystalline structure [16, 17].

Most of the studies demonstrating the effect of CO_2_-laser and fluoride therapy on solubility or microhardness enhancement of enamel are in vitro [5, 12–14, 16, 18]. Few clinical studies investigating the efficacy of this newly developed laser irradiation pattern in the prevention of dental caries are retrieved [19, 20]. Therefore, the investigation of this synergetic effect of laser irradiation and topical fluoride application could be a good prophylaxis option and will improve the quality of dental care.

### Objectives

The aim of the study is to assess the viability and efficacy of the CO_2_-laser in combination with a fluoride varnish in the prevention, severity, and extent of white spot lesions during orthodontic treatment with fixed appliances in a randomized, open-label, split-mouth, controlled, clinical trial over 12 months. The mineral loss will be quantified by visual examinations with the International Caries Detection and Assessment System (ICDAS) and SoproLife® Daylight and Blue fluorescence.

### Trial design

The research is designed as a longitudinal randomized controlled clinical cross-over experimental study with a split-mouth design. This type of trial reduces the variability in trial outcomes, but patient recruitment is too difficult and often takes more time than expected. Table 1 presents the recruiting, allocation, interventions, monitoring, and analysis of the research in accordance with the Standard Protocol Items: Recommendations for Interventional Trials recommendations [21].

**Table 1:** Trial design. The table summarizes the enrolment, allocation, interventions, and assessments in the trail

	Study period						
	Enrolment	Allocation	Post-allocation				
**Timepoint**^**a**^	**−** ***t***_**1**_	**0**	***t***_**1**_	***t***_**2**_	***t***_**3**_	***t***_**4**_	***t***_**5**_
**Enrolment**							
Eligibility screening	**×**						
Informed consent	**×**						
Allocation		**×**					
**Interventions**							
Topical fluoride application + CO_2_-laser irradiation				**×**			
Topical fluoride application (placebo manipulation)				**×**		**×**	**×**
Bonding brackets					**×**		
**Assessments**							
ICDAS score			**×**			**×**	**×**
*SoproLife*® Daylight fluorescence			**×**			**×**	**×**
*SoproLife*® Blue fluorescence			**×**			**×**	**×**

^a^Post-allocation time frame: *t*1, before the start of the treatment; *t*_2_, during laser irradiation/placebo manipulation; *t*_3_, orthodontic treatment; *t*_4_, 6 months after the laser irradiation, *t*_5_, 12 months after the laser irradiation

Due to the nature of the interventions, the doctor, dental assistant, and the patient are not blinded and the trial is an unmasked, open-label trial. The experimental manipulation includes topical fluoride application and CO_2_-laser irradiation of the left maxillary anterior teeth and topical fluoride application (placebo manipulation) to the contralateral teeth of the same patient.

## Methods and analyses

### Study setting

The study setting of this research includes the Department of Paediatric Dentistry and the Laser Centre of the Faculty of Dental Medicine, Medical University – Plovdiv, Bulgaria.

### Eligibility

#### Inclusion criteria

1. Participants in the study are 12–18-year-old children, in good general health;
2. Children, requiring fixed orthodontic treatment (fixed braces);
3. Children at high caries risk according to “Oral disease risk assessment tool” accepted and used in the Department of Paediatric Dentistry, Faculty of Dental medicine, Medical University – Plovdiv, Bulgaria (see supplementary data file S1 “Appendix for risk assessment of oral diseases”);
4. Children with untreated non-carious vestibular surfaces of the six maxillary anterior teeth of the permanent dentition (central and lateral incisors, canines) with ICDAS code 0 [22–24];
5. Verbal assent from the child willing to comply with all study procedures and protocol; and
6. Obtained written informed consent by the patient’s parent/guardian for participation in the study (see supplementary data file S2 “Patient consent form” and S3 “Information leaflet”).

#### Exclusion criteria

1. Children with systemic diseases that may affect oral health or oral microflora (such as diabetes);
2. Children with medication intake that could affect the salivary flow or oral microflora (such as antibiotics);
3. Children with mental or cognitive problems; and
4. Patients with maxillary anterior permanent teeth affected by disturbances in the development of dental structures (hypoplasia, hypomineralization, fluorosis).

#### Interventions

A low-speed rubber cup and pumice paste (CleanPolish, Kerr) will be used for 30 s for cleaning and polishing of the maxillary anterior teeth and then they will be washed for debris and organic residue removal. A baseline visual inspection using ICDAS criteria of the vestibular surfaces of the investigated teeth as well as white and blue light digital photographs using the device SoproLife® (Sopro-Acteon group, La Ciotat, France) will be made.

##### Topical fluoride application protocol

(according to the instructions for use of the manufacturer):

- Following the instructions of the dosing guide, the required amount of the varnish (Clinpro™ White Varnish 5% Sodium Fluoride, 3 M ESPE, USA) is determined—0.5 ml
- Isolation
- Opening of the unit-dose package of the varnish and dispensation of the entire content onto the round dosing guide on the back of the foil pouch follows
- Since components of all sodium fluoride varnishes can separate during storage, thoroughly mixing with the applicator brush is required
- Application of the varnish evenly in a thin layer over the whole vestibular surfaces of all anterior maxillary teeth with sweeping, horizontal brush strokes. No suction is required

##### Laser irradiation protocol

The vestibular surfaces of the left maxillary anterior teeth will be exposed to CO_2_-laser (Ultra Dream Pulse, DS_40U, Daeshin Enterprise, Seoul, South Korea), emission wavelength 10,600 nm. The parameter settings used will be:

Time on—100 μs, time off—40 ms;

Average power—0.73 W; peak power—292.73 W;

Speed of movement—2 mm/s;

Energy density with movements—5 J/cm^2^;

Tip-to-tissue distance—20 mm; tip diameter 700 μm; and

Irradiation time—30 s.

The measured values were confirmed using a power meter. The chosen protocol parameters are modified in a previously conducted study [5].

##### Placebo protocol

The vestibular surfaces of the right maxillary anterior teeth are exposed to sham light (as a placebo light). No pulse energy will be applied.

##### Bonding brackets protocol

*Bonding brackets protocol* includes the following steps:

- Isolation
- Tooth surfaces will be etched with 35% phosphoric acid gel (Etching gel, DMP Ltd.) for 30 s and rinsed for the same time
- Reisolation
- Tooth surfaces will be dried with air spray for 15 s
- An adhesive bonding agent (AdperTM single bond, 3 M ESPE, USA) will be applied on the enamel surfaces according to the manufacturer’s instruction, and then will be cured for 20 s
- Fluoride-free Transbond XT resin composite (3 M Unitek, USA) will be applied, and a stainless steel orthodontic bracket with a slot size of 22 (3 M Unitek, USA) will be placed while excess composite will be removed. Resin composites will be cured for 20 s of occlusal, gingival, mesial, and distal directions.

The patient will be recalled 6 and 12 months post-irradiation. A visual inspection and reapplication of the fluoride varnish will be conducted by the same operator. During the evaluation, the teeth will be thoroughly cleaned with a rubber cup and pumice and then washed. After drying, the vestibular surfaces will be visually evaluated using ICDAS and SoproLife® daylight and blue fluorescence, followed by topical fluoride application.

#### Clinical protocol

First visit:

1. Parents/guardians are informed about the protocol of the study and the laser technique. They sign the informed consent form (see Supplementary data file S2 “Patient consent form”). Verbal assent from the child is obtained.
2. The teeth are cleaned and polished with a rubber cup and pumice paste.
3. A visual inspection of the vestibular surfaces of the investigated teeth and SoproLife® Daylight and SoproLife® Blue fluorescence digital images are made and recorded.
4. Application of fluoride varnish Clinpro™ White Varnish 5% Sodium Fluoride over the whole vestibular surfaces of all anterior maxillary teeth
5. The chosen method, CO_2_-laser irradiation or placebo, is applied.
6. Follows direct adhesive bonding of orthodontic brackets

Second visit:

1. The teeth are cleaned and polished with a rubber cup and pumice paste.
2. The incidence, extent, and severity of the lesions are assessed using ICDAS criteria and SoproLife® daylight and blue fluorescence. The data are recorded and digital photographs are made.
3. Reapplication of the fluoride varnish Clinpro™ White Varnish 5% Sodium Fluoride

Third visit:

1. The teeth are cleaned and polished with a rubber cup and pumice paste.
2. The incidence, extent, and severity of the lesions are assessed using ICDAS criteria and SoproLife® daylight and blue fluorescence. The data are recorded and digital photographs are made.
3. Reapplication of the fluoride varnish Clinpro™ White Varnish 5% Sodium Fluoride

#### Criteria for discontinuing or modifying allocated interventions

1. The patient withdraws consent
2. Inter-current illness preventing further treatment

### Outcomes

#### Primary outcome measures

The primary outcome measures are the number of newly appeared caries lesions and the changes into ICDAS scores in the experimental and control teeth (within one patient) from baseline to 12 months. The International Caries and Detection System (ICDAS) is a visual scoring system developed for use in clinical assessment and clinical research of caries development and progression. It uses a numbered scoring system with a range from 0 to 6, to grade enamel demineralization and cavitation. Code of “0” represents unaffected/sound enamel, no caries change; “1” first visual change represents initial demineralization that is visible only after air drying; “2” represents a distinct visual change in the enamel that is visible when the surface is wet; “3” represents localized enamel loss or breakdown, no visible dentin or underlying shadow; and “4-6” represent larger carious lesions in dentin. Time frame: 1 year.

#### Secondary outcome measures

The secondary outcome measures are changes in SoproLife® scores in the experimental and control teeth (within one patient) from baseline to 12 months. The SOPROlife score is a visual assessment of the caries levels. The SoproLife® daylight intraoral camera (SoproLife® Daylight mode) is used to record an intraoral picture with Sopro-imaging software (Acteon, Sopro, La Ciotat, France). The SoproLife® blue fluorescence mode scores range from 0 to 5. Code of “0” is given when the surface appears shiny green, the enamel appears sound, and there are no visible changes’ code “1” a tiny, thin red shimmer is observed, no red dots appeared; code “2” darker red spots are visible; code “3” dark red spots have extended, a slight beginning roughness of the red areas can be visible; and codes “4–6” represent larger carious lesions in dentin. Time frame: 1 year.

#### Participant’s timeline

Each eligible patient undergoes three visits. The first appointment includes screening, consenting and assenting, recording of initial values, topical fluoride application, and treatment with the selected specific parameters of CO_2_-laser of the left maxillary anterior teeth and Placebo procedure for the right, contralateral teeth, and orthodontic treatment at the end of the visit. The second appointment at the 6-month recall includes an oral examination, cleaning of the investigated surfaces, collection of data values, reapplication of the fluoride varnish. The third appointment at the 12-month recall includes an oral examination, cleaning of the investigated surfaces, collection of data values, reapplication of the fluoride varnish.

#### Sample size calculation

The sample size calculation is based on our primary outcome endpoint of changes in ICDAS score on the vestibular surfaces of the anterior maxillary teeth in the left and the right quadrants per subject, by comparing the proportion of teeth with worsening ICDAS score in experimental in comparison to the control group. To determine the sample size for each group, a priori power analysis was conducted as follows:

$$ n=\frac{\left(z\frac{\upalpha}{2}+\mathrm{z}\upbeta \right)2\left(p1\left(1-p1\right)+p2\ \left(1-p2\ \right)\right)}{\begin{array}{c}\left(p1-p2\right)2\\ {}\end{array}} $$

*p*1: 8.7% of the lesion incidence in the control group

*p*2: 3.6% of the lesion incidence in the laser group

The data for this power analysis were obtained from a previous study.^9^ The significance level was considered as 95% and power was 80%. By inserting the minimum values in the above formula, the sample size was calculated as 241 teeth (121 teeth per group, i.e., a total number of 41 patients).

### Recruitment

The patients at the Department of Paediatric Dentistry of the Faculty of Dental Medicine, Medical University – Plovdiv, Bulgaria, who meet the inclusion criteria, will be screened for eligibility. Once identified, patients will be informed about this research project and will receive information about the possibility of potential study participation. Patient recruitment starts obtaining the full quota of participants within a 1-year time frame. It begins in September 2021 with an estimated enrollment capacity of 4 patients per month.

### Participating centers

The patients are randomly selected from the visitors in the Department of Paediatric Dentistry of the Faculty of Dental Medicine, Medical University – Plovdiv, Bulgaria, and treated in the Laser Centre of the same university.

### Assignment of the intervention

#### Sequence generation

A randomization sequence for the allocation of the quadrants will be created using Random Allocation Software. The study is a split-mouth randomized control study and each patient will receive both procedures during the first visit. The patient will be randomized to receive first the laser irradiation and second the placebo procedure or first the placebo procedure and second the laser irradiation before the bonding of the brackets.

#### Allocation concealment mechanism and implementation

The sequence of the application of the two protocols, laser and placebo, is randomly assigned. A randomization list will be created by a random generator and kept in a locked drawer. Assignments will be kept in separate, closed opaque, sequentially numbered envelopes, enabling the sequence to be concealed until the intervention is assigned.

#### Blinding

As participants in the treatment as well as the nature of the intervention, the operator and the dental assistant will not be blinded to subjects’ group assignments. The patient will be also not blind to the treatment. The dental assistant will inform the doctor which protocol will be the first to start with and which will be the second protocol. The statistician will be blinded to treatment assignment as data will be masked before the analysis by labeling the 2 quadrants as 0 and 1, without giving the statistician the key.

#### Data collection, confidentiality, storage, and monitoring of the study documents

Collection, coding, storage, and evaluation of personal data within the project will be carried out in accordance with The General Data Protection Regulation (EU) 2016/679 (GDPR). A prerequisite for data collection will be the voluntary written informed consent of the patient’s parent or guardian. Confidentiality will be guaranteed by a coded ID number on the paper-based form, access will be granted exclusively to the study investigators. The information from the paper forms will be exported to a database file by using only the ID number and stored on a password-protected computer. Only the investigators and the statistician will have access to the final data set. All data collected on paper-based forms will be stored in sealed containers in areas of the Department of Paediatric Dentistry, Faculty of Dental Medicine, Medical University – Plovdiv, Bulgaria with limited access for 10 years in line with the Medical University of Plovdiv’s research procedures. The data custodian will be the MS.

## Statistical methods

The obtained data will be recorded, tabulated, processed, and analyzed using SPSS (Statistical Package for Social Science software) version 21.0 (IBM, USA). Descriptive statistics will be calculated. For the split-mouth randomized controlled trial Student t-test for paired samples will be used for the analysis. Mixed effects proportional odds regression modeling with outcomes of ICDAS and SOPROLIFE scores will be calculated. The inter-observer reliability and agreement will be estimated by Kappa coefficient where Kappa coefficient values above 0.80 are considered acceptable. Missing Completely at Random (MCAR) will be used to handle the missing data.

## Patient and public involvement

The development of the research question and outcome measures will be based on the review of available evidence in this research area. Patients will not be involved in the development of the study protocol. During the conduction of the study, patients will not be informed about the results of the ongoing trial since there is no planned interim analysis. The results will be disseminated to the study participants through email and routine follow-up dental check-ups.

## Ethics and dissemination

The clinical study will be conducted in accordance with the conditions and principles of the Declaration of Helsinki, the existing EU Clinical Trial Directive (EC) No. 2001/20/EC, the recommendations of the Ethical Committee at the Medical University of Plovdiv, Bulgaria, and the international ethical and scientific quality standard for designing, recording and reporting trials that involve the participation of human subjects - Good Clinical Practices (GCP).

### Research ethics approval

The study was approved by the Committee for Scientific Research Ethics, Medical University - Plovdiv, Bulgaria (Reference number P-605/27.03.2020, Protocol of approval No. 2/01.04.2021) and registered on a publicly accessible database ClinicalTrials.gov (Registration number: NCT03412721). Ethical approval for the study protocol and the written informed consent for all subjects’ parents/guardians was granted by the Ethics Committee of the Medical University, Plovdiv, Bulgaria.

### Consent

The operators will obtain written consent from patients’ parents/guardians willing to participate in the trial. Additional information will be provided for all parents for the study. Completed informed consent will be collected at the Department of Paediatric Dentistry, Medical University - Plovdiv by the study investigators. A copy of the signed consent form will be handed over to the participating child’s parent/guardian. After providing age-appropriate information about the study, verbal assent will be obtained as an affirmative agreement for participation from children

### Confidentiality

The information of the participants collected during the study will be kept strictly confidential and will not be disclosed to third parties. Confidentiality will be guaranteed by a coded ID number, access will be granted exclusively to the study investigators. During the planning phase of a clinical trial Medical University of Plovdiv, Bulgaria, the clinical trial sponsor, assessed that Data Monitoring Committee (DMC) was not needed for this clinical trial. As described above, a non-life-threatening procedure is the objective of this study as well as the second and third visits will be with very short duration in different timepoints for each patient. Due to this reason, the use of a DMC might not be beneficial for the study but might even delay the finalization of the trial.

### Conflict of interests

The investigators have no conflicts of interest to declare. They agree with the protocol and the informed consent of the study and there is no financial interest to report.

### Access to data

All data collected will be stored in sealed containers in areas of the Department of Paediatric Dentistry, Faculty of Dental Medicine, Medical University – Plovdiv, Bulgaria with limited access. The information from the paper forms will be exported to a database file and stored on a password-protected computer. Only the investigators and the statistician will have access to the final data set. The quality of the clinical trial, conducted by the investigators, will be assessed by the Research Committee of Medical University of Plovdiv’s audits and regulatory inspections every 6 months.

### Dissemination policy

The results of the trial will be presented through peer-reviewed publications and conference presentations. In addition, our results will be disseminated to clinicians, as well as key stakeholders, including scientific directors of postgraduate programs “Master of Science in Lasers in Dentistry,” academic courses in Pedodontics and Preventive dentistry.

## Conclusion

This randomized control trial is a well-powered, one-center split-mouth experimental study. In split-mouth designs, experimental and control interventions are applied to different areas in the same oral cavity and the advantage is the reduction of the outcome variability estimation, leading to the potential increase in statistical power.

Fixed orthodontic treatment is associated with a high risk of initiation and development of caries lesions. CO_2_-laser has great potential in dental caries prevention. The study outlined in this protocol will be the first direct investigated combination of the preventive effect of the CO_2_-laser irradiation in addition to fluoride therapy and the fixed orthodontic treatment as a risk factor for dental caries. The implementation of CO_2_-laser in the regular protocol for the orthodontic treatment would significantly increase the success of this therapy resulting in lower rates of white spot lesions associated with it.

## Trial status

The trial is not yet recruiting patients. The process will start in September 2021 and the follow-up phase of the trial will continue until September 2023.

## Supplementary Information

**Additional file 1.** Appendix for risk assessment of oral diseases.

**Additional file 2.** Patient consent form.

**Additional file 3.** Information leaflet.

**Additional file 4.** Statement of the Committee for Scientific Research Ethics of Medical University – Plovdiv, Bulgaria.

## Data Availability

Data available within the article or its supplementary data materials.

## Abbreviations

CO_2_: Carbon dioxide
CaF_2_: Calcium fluoride
ICDAS: International Caries Detection and Assessment System
LASER: Light Amplification By Stimulated Emission Of Radiation
SPIRIT: Standard Protocol Items for Randomized Trials
