# Supplementary material for: Caries inhibition with CO2-laser during orthodontic treatment: a study protocol for a randomized split-mouth controlled clinical trial
Source: Trials. 2022 Mar 12;23:208. doi: 10.1186/s13063-022-06117-y (PMC8917669; doi:10.1186/s13063-022-06117-y)
Supplement: Supplementary file 1 — Additional file 1. Appendix for risk assessment of oral diseases. [file 13063_2022_6117_MOESM1_ESM.pdf]

APPENDIX FOR RISK ASSESSMENT OF ORAL DISEASES

RISK FACTORS	RISK INDICATORS		
**I. Part: Anamnesis**	**HIGH**	**AVERAGE**	**LOW**
Medically compromised patient	**Yes**	**-**	**No**
Factors associated with decreased salivary flow	**Yes**	**-**	**No**
Dental examinations of oral health	**No**	**Irregular**	**Regular**
Existing carious lesions	**Yes**	**-**	**No**
New carious lesions	**< 12 months**	**12-24 months**	**> 24 months**
Fixed/removable orthodontic appliance	**Yes**	**-**	**No**
Parents with untreated carious lesions	**Yes**	**-**	**No**
Social status	**Low**	**Average**	**High**
Daily intake of carbohydrate foods and drinks	**>3**	**1-2**	**Only with main meals**
Fluoride intake	**No**	**Only from fluoride toothpaste**	**Fluoride toothpaste, mineral water, fluoride supplements**
Frequency of oral hygiene	**< 1 a day**	**1 a day**	**1-3 times a day**
**II. Part: Clinical examination**			
Visible plaque	**Yes**	**-**	**No**
Gingivitis	**Yes**	**-**	**No**
Areas with demineralization of the enamel	**>1**	**1**	**None**
Enamel defects, deep pits, and fissures	**Yes**	**-**	**No**
**III. Part: Paraclinical examinations**			
Radiologically visible enamel lesions	**Yes**	**-**	**No**
Levels of Str. mutans, Lactobacilli	**High**	**Average**	**Low**
**The presence of even one risk indicator in the "average" or "high" risk categories means the patient belongs to the respective group.**			

Conclusion based on the risk assessment of oral diseases:

....

....

....
