# Supplementary material for: Caries inhibition with CO2-laser during orthodontic treatment: a study protocol for a randomized split-mouth controlled clinical trial
Source: Trials. 2022 Mar 12;23:208. doi: 10.1186/s13063-022-06117-y (PMC8917669; doi:10.1186/s13063-022-06117-y)
Supplement: Supplementary file 2 — Additional file 2. Patient consent form. [file 13063_2022_6117_MOESM2_ESM.pdf]

PATIENT CONSENT FORM

DEPARTMENT OF PAEDIATRIC DENTISTRY
LASER CENTER
FACULTY OF DENTAL MEDICINE,
MEDICAL UNIVERSITY OF PLOVDIV, BULGARIA

Name of patient: ______________________________________

Patient`s parent/guardian: ______________________________________

Material – text (information about your child`s oral health) and digital photographs (intraoral digital photographs using the device SoproLife ®) ______________________________________

Provisional title of article in which Material will be included: Caries inhibition with CO_2-laser during orthodontic treatment: a study protocol for a randomized split-mouth controlled clinical trial

---

CONSENT

I____*[YOUR FULL NAME]* give my consent for the Material about my child to appear in a publication.

I confirm that I: *(please tick boxes to confirm)*

- ☐ ***have seen the images and text or other material about my child***
- ☐ ***have read the article to be submitted to a journal***
- ☐ ***am legally entitled to give this consent.***

I understand the following:

(1) The Material will be published without my child`s name attached, however I understand that complete anonymity cannot be guaranteed. It is possible that somebody somewhere may recognise my child.

(2) The Material may show or include details of my child`s medical condition or injury and any prognosis, treatment or surgery that the child has, had or may have in the future.

(3) The article may be published in a journal which is distributed worldwide.

(4) The article, including the Material, may be the subject of a press release, and may be linked to from social media and/or used in other promotional activities.

(5) The text of the article will be edited for style, grammar and consistency before publication.

(6) I and my child will not receive any financial benefit from publication of the article.

(7) The article may also be used in full or in part in other publications and products. This includes publication in English and in translation, in print, in digital formats, and in any other formats that may be used by publishers now and in the future. The article may appear in local editions of journals or other publications, published in the UK and overseas.

(8) I can revoke my consent at any time before publication, but once the article has been committed to publication it will not be possible to revoke the consent.

(9) This consent form will be retained securely in accordance with the law, for no longer than necessary. Personal data provided in this form will be used and retained in accordance with journal`s Privacy Policy.

Signed:* ______________________________

Parent/Guardian`s name: ______________________

Address: ______________________________

____

Email address: ______________________________

Telephone no: ______________________________

** signing on behalf of the patient who is under the age of 18*

______________________________ *Date:* ______________________________

Corresponding author

*Signed:*______________________________

Author`s name: Maria Shindova

Position: Senior Assistant Professor

Institution: Department of Pediatric Dentistry, Faculty of Dental Medicine, Medical University of Plovdiv

Address: 3 Hristo Botev Bulv., Plovdiv, Bulgaria

Email address: mariya.shindova@gmail.com

Telephone no: + 359 898 390 935

Date: ______________________________
