# Supplementary material for: Caries inhibition with CO2-laser during orthodontic treatment: a study protocol for a randomized split-mouth controlled clinical trial
Source: Trials. 2022 Mar 12;23:208. doi: 10.1186/s13063-022-06117-y (PMC8917669; doi:10.1186/s13063-022-06117-y)
Supplement: Supplementary file 3 — Additional file 3. Information leaflet. [file 13063_2022_6117_MOESM3_ESM.pdf]

INFORMATION LEAFLET

DEPARTMENT OF PAEDIATRIC DENTISTRY
LASER CENTER

FACULTY OF DENTAL MEDICINE,
MEDICAL UNIVERSITY OF PLOVDIV, BULGARIA

CARIES INHIBITION WITH CO_2-LASER DURING ORTHODONTIC TREATMENT

DESCRIPTION: You and your child are invited to participate in a research study on the efficiency of CO_2-laser in combination with a fluoride varnish in the prevention of caries lesions during orthodontic treatment with fixed appliances.

PROCEDURES: With your permission, we would like to collect information about your children`s oral health at the beginning and during the process of the orthodontic treatment with fixed appliances. Also, we would like to collect information about the incidence and severity of new caries lesions after the start of the orthodontic treatment as well as monitoring the caries-preventive effect of the laser irradiation and the application of fluoride varnish. This study does not involve any experiments, just preventive procedures and orthodontic treatment, collection, and study of the required information.

RISKS AND BENEFITS: There are no anticipated risks associated with this study. You will not receive any direct benefit from participation.

TIME INVOLVEMENT: Your child`s participation in this study will not require more time from you other than for the first visit including an explanation of the study, oral examination, preventive procedures (laser irradiation, and application of fluoride varnish), and orthodontic treatment. The second and the third appointments at 6-and 12-month recall will include professional oral hygiene, oral examination, and reapplication of the fluoride varnish.

PAYMENTS: You will not be paid to participate in this study. You will not pay for the treatment of your child in this study.

PARTICIPANT'S RIGHTS: If you have read this form and have decided your child to participate in this research, please understand your participation is voluntary and you have the right to withdraw your consent or discontinue participation at any time without penalty or loss of benefits to which you are otherwise entitled.

The results of this research study may be presented at scientific or professional meetings or published in scientific journals. However, your identity will not be disclosed**.**

Thank you for your time and attention!

Name of parent/guardian ....

Signature of parent /guardian ....

Name of patient ....

Telephone number ....

Name of the dentist ....

Signature of the dentist ....

For additional information regarding the trial, you can contact us at the given address, emails, or phone numbers.

Researchers:

Maria Shindova, DDS, MSc, PhD
Chief Assistant Professor
Department of Paediatric Dentistry
Faculty of Dental Medicine
Medical University – Plovdiv
3 Hristo Botev Blvd
4000 Plovdiv, Bulgaria
Mobile: + 359 898 390 935
mariya.shindova@gmail.com
Mariya.Shindova@mu-plovdiv.bg

Ani Belcheva, DDS, MSc, PhD
Professor
Department of Paediatric Dentistry
Faculty of Dental Medicine
Medical University - Plovdiv
3 Hristo Botev Blvd
4000 Plovdiv, Bulgaria
Mobile: + 359 889 528 932
abeltcheva@yahoo.com
