# Supplementary material for: Caries inhibition with CO2-laser during orthodontic treatment: a study protocol for a randomized split-mouth controlled clinical trial
Source: Trials. 2022 Mar 12;23:208. doi: 10.1186/s13063-022-06117-y (PMC8917669; doi:10.1186/s13063-022-06117-y)
Supplement: Supplementary file 4 — Additional file 4. Statement of the Committee for Scientific Research Ethics of Medical University – Plovdiv, Bulgaria. [file 13063_2022_6117_MOESM4_ESM.pdf]

STATEMENT
of the Committee for Scientific Research Ethics
of Medical University – Plovdiv, Bulgaria

Concerning/To: Rector`s order № 605/27.03.2020: Clinical trial of as.prof. Maria Shindova, DMD, PhD and prof. Ani Belcheva, DMD, PhD in Pediatric dentistry in the Department of Pediatric Dentistry, Faculty of Dental Medicine, Medical University-Plovdiv, Bulgaria, title `Emotional and behavioural adaptation of children to dental treatment`.

After review and detailed consideration of the presented documentation (including Abstract, Consent form, Parental authorization form, Patient`s inquiry card) at a meeting of the Committee for Scientific Research Ethics, Medical University-Plovdiv, Bulgaria (protocol Reference number №2/01.04.2021), the Committee considers:

The present scientific research corresponds to the scientific and ethical standards and criteria in research and the requirements of: Declaration of Helsinki - Ethical Principles for Medical Research Involving Human Subjects, Principles of Good Clinical Practice, Bulgarian legislation and regulation for clinical and scientific research involving human subjects.

Date: 01.04.2021
